# Astrobiology - an opposing view

**DOI:** 10.6026/97320630014346

**Published:** 2018-06-30

**Authors:** Paul Shapshak

**Affiliations:** 1 Division of Infectious Diseases and International Health, Department of Internal Medicine, University of South Florida, Morsani College of Medicine, Tampa, FL 33606, USA

**Keywords:** Artificial Intelligence, quantum computers, astrobiology, exobiology, life, origin, ecosystem, Archaea, bacteria, viruses, space exploration, exoplanets, moons, asteroids, comets, goldilocks zone, methane, greenhouse gasses, haze, smog, NASA, ESA, crashed spacecraft, rovers, solar system contamination by terrestrial organisms, chirality, diasteriomer

## Abstract

The use of quantum computers and Artificial Intelligence (AI) is imperative for use in space exploration and astrobiology investigations. Considerable progress has been made since the commencement of origin of life laboratory and theoretical studies in the mid 20th century. However, the sheer amount of data amassed to date in all these studies including exoplanetary and astrobiological studies is enormous and increasing steadily. Thus, there is the need for AI and quantum computers. As AI develops, it will become crucial in the development of the statistical and database programs that are indispensable to analyze the huge quantity of cumulative data. Diverse biotic and geochemical processes have been shown to produce methane on the Earth. Elsewhere in the solar system, on other planets (e.g. Mars) and moons (e.g. Titan), as well as on exoplanets, abiotic processes are considered the primary sources of methane. Astronomers and astro-biologists infer that the presence of methane supports the possibility of the presence of at least microbial life. In addition, on the Earth, there are also degradative reactions that include smog-related compounds and hazes that are produced as artefacts of intrinsic methane geochemistry as well as due to human footprint. Astronomers and astro-biologists envision life, away from the Earth, elsewhere in the solar system and on exoplanets, to occur under conditions similar or related to terrestrial life (goldilocks zone) conditions. These properties that are compatible with life as we know it on the Earth, include planetary orbits, gravitation, star radiant energy, presence of liquid water, and compatible temperatures and pressures, found on Earth. Generally, extraterrestrial life is also considered to resemble the biochemistry, molecular biology, and physiology of life on Earth - thus the focus on detection of supposed biosignatures of microbial life that resemble the Earth's. Nevertheless a crucial factor is absent in these deliberations - viruses. On the Earth, viruses that infect Archaea and bacteria form local and widespread global ecosystems. These viruses play a crucial role and facilitate the molecular transfer of host genes among various hosts. This essential function is underestimated in evolutionary as well as astrobiological speculations. Thus, it is of substantial importance to consider the roles that viruses may have played during the origin of life as well as in any exobiology.

## Background

Kasting and colleagues reported in 2010 that more than 300 exoplanets were detected [1]. Remarkably, as of June 2018, 3,786 exoplanets have been catalogued [2]. Of these, 53 habitable exoplanets occur in goldilocks zones [3]. Refined spectroscopic measurements identified gases on these exoplanets including CH4, H2O, N2O, O3, and CO2. The complexities of the progression of CH4 and N2 levels on the Earth and Venus are under continued study. It is hypothesized that prebiotic conditions favored N2 and CO2 on the primitive Earth, CH4 accumulated after the origin of methanogenic bacteria, and this also resulted in hazes on the primitive Earth. Currently, on Titan, a moon of Saturn's, atmospheric haze and clouds are also considered due to chemical reactions involving CH4 [1, 4].

It should be noted that early work on taxonomy contends that Archaea are one of three domains of the roots of extant terrestrial life and that the other two include Bacteria and Eukarya [5]. However, there is disagreement on this issue. If more than the naive use of the16S ribosomal RNA sequence evolutionary tree is included in the analysis of the various individual and group properties of the three domains, then it is contended that Archaea and bacteria are the two primary domains of terrestrial life. A variety of evidence supports the notion that Eukarya are descended from both Archaea and bacteria [6].

Viruses are generally ignored in questions of association of terrestrial microorganisms and methane. However, Archaea, bacteria, and their infecting viruses and phage form local ecosystems that are also components of the larger terrestrial global ecosystem. Moreover, viruses infect one third of the ocean microbiome and have the capacity to transfer host genes among microorganisms. This has an impact on each particular niche that viruses and their hosts occupy, evolution of their ecosystems, and results in environmental transformation. Consequently, the terrestrial virospheres (that includes viruses and phages) of Archaea and bacteria are substantial and their roles in ecology necessitate further study [7, 8].

### Methane

It is generally hypothesized that methanogen bacteria were the dominant microbial life forms on Earth prior to 2.3 billion years ago (bya) [7]. Various conditions that permitted the origin of this type of life and promoted growth of such microorganisms include haze, 30% dimmer Sun, and lack of oxygen and ammonia [7]. At these earlier times, current theories rely on a methane dominant model atmosphere with a type of greenhouse effect to counteract the lower solar luminosity, which would have otherwise turned the Earth into an ice planet. It is considered probable that methanogens on the early Earth produced 600 times more methane than they do today [7, 8, 9, 10, 11]. After 2.3 bya, organisms evolved and prevailed, which caused a change from a predominantly reducing atmosphere to a predominantly oxygencontaining atmosphere. It should be noted that oxygen conditions are not amenable to early Archaeal life on the Earth, although Archaeal microbiota survive contemporaneously in various ecological niches including hydrothermal vents and fissures on the deep ocean floor [7, 8].

The equilibrium and non-equilibrium dynamics of CH4, NH3, O2, and CO2 during the evolution of Earth, Venus, and Mars are hotly debated. Data concordance, though, is reported lacking. However, it is concluded that the presence of CH4 or O2 would strongly support the existence of life, similar to what we know, on exoplanets that have 'goldilocks' or Earth-akin conditions [4, 12]. On this issue, further studies produced in situ measurements on Mars that confirmed prior off-surface observations. Most recently, in situ studies by the rover, 'Curiosity', during 38 months, confirmed low levels of CH4 in the Mars atmosphere, with seasonal fluctuations at or near the Gale crater. Peak production occurs at a solar longitude of 160 degrees, which is during Mars' northern hemisphere summer. Various geomorphological rationales and hypotheses were deliberated as well, in the article [13].

An additional model for CH4 production involves iron- and nickel-containing asteroid impacts that fell to the primitive Earth. Fischer-Tropsch-type reactions leading to methane production could occur when asteroids impact the Earth's atmosphere. Interestingly, different models are provided for the application of the Fischer-Tropsch reaction to nickel- and iron-containing asteroids. Proposed alternatives include methane production within asteroids, methane production externally, at the time that they impact the Earth's atmosphere, or later as the atmospheric condensates, due to asteroid impacts, fall to Earth [14, 15].

To explain fluctuating methane levels on Mars and Titan, abiotic means of replenishing methane are hypothesized. However, possible hypothetical biotic mechanisms are promulgated as well [16]. We note, contrariwise, that since the existence of extraterrestrial life itself is an improbable event, it would seem unlikely that biotic processes are a cause d'etre for the presence and fluctuations of extraterrestrial methane.

### Greenhouse processes

Hypothetical early climatic histories of Earth and Mars have been compared for some time. Solar flux and greenhouse effects due to chemistry of gases including CH4, CO2, and H2O are under extensive analysis. It is concluded that there are sufficient greenhouse effects on planets that would otherwise be colder, due to their distances from their stars. Rather than concluding that Earth-like life may have arisen, we infer that such planets may be habitable by terrestrial life [2, 13, 14, 15].

The detection of haze on Titan and possibly on exoplanets is speculated to be a possible biosignature and due to a variety of molecules that include carbon and nitrogen. Detection of haze over the Indian ocean and smog in very large cities around the globe are considered to be the results of UV interactions with pollutants and greenhouse gases including CO2, CH4, N2O, CO, NOx, and SO2. Burning fossil fuels including coal and natural gas predominantly produces these gasses, fumes, and soot. Deforestation due to slash- and burn-culture, results in further spreading these deleterious conditions [16, 17, 18, 19, 20, 21, 22].

### Is there an Astrobiology?

The fundamental canonical equation and algorithm used in Astrobiology is the Drake equation. In recent astrobiological studies, some of the inputs related to the existence of life underwent further examination. What is demonstrably incongruous and circular reasoning, applied to the Drake equation, is to use a factor of 100% for the probability of detecting simple exobiological microbes because it is postulated to be at that elevated level of probability for goldilocks zone exoplanets [23]. Such calculations require additional study.

Lineweaver and Davis performed calculations of probabilities of Exobiology. In brief summary, they state that there is geological evidence that life arose on Earth during the first 200 million years (Myr) or at least upto the first billion years [24]. Based on a statistical lottery model for goldilocks zone planets, they calculate a probability of 36% for the occurrence of life on such planets that are older than 1Gyr. However, their calculations can be in error if their assumptions and events selected in building the model are incorrect. Moreover, in further contradistinction, they point out that rapid biogenesis may be implausible for their model. An additional publication further indicates the problematic assumptions in this model and their probability reduced to 13% [25].

There was an initial cooler period, less than 1Gyr, for the early Earth to accrete and grow. Subsequently, there was an increase in temperature upto 3,000 oC for various melting and differentiating processes to have occurred (Hadean epoch), with subsequent cooling, resulting in the Earth's mantle as we find it today [26]. It should be noted that there was much greater radioactivity during the earlier period. Thus, living organisms would have had to find some sort of shielding to escape their destruction due to radioactivity.

Strikingly, no rocks or relics remain from that cooler purportedly more hospitable early period of the Earth. A subsequent Hadean period followed with high temperatures as mentioned, which must have obliterated all prior cooler materials. From the earliest Earth, only a few single xenocrystic zircons have been found in Western Australia. These few small crystals mineral fragments remain the sole evidence of conditions from that time, supporting the existence of the hypothesized brief cooler period antecedent to the Hadean epochs [27]. Improved definition and data are needed as to the fossil evidence for the origin of life during the early hypothesized goldilocks Earth vis a vis Hadean Earth's multiple annihilation and extinction events. Consequently, the null hypothesis remains tenable that life is improbable.

## Conclusions

From the start of origin of life experimentation, the goldilocks paradigm and hypothesis (centered on Earth-like conditions) has gripped the outlook on ascertaining the chemical and environmental conditions necessary for the origin of life. The focus is on a limited number of compounds as mentioned. [16] Key processes may be missed if alternatives are not simultaneously explored related to the origin of life, besides the question of habitability. Moreover, strict criteria of data vs. speculations that appear appealing need be distinguished.

On the one hand, it is speculated that goldilocks environmental conditions promote the origin of life. However, on the other hand the hadean effects of haze and smog production are not proposed as biosignatures of intelligent technological life. Besides, the use of spacecraft, rovers, and other observational methods for space exploration are sufficient rationales for an understanding of planetary sciences without using the unlikely event of life elsewhere in the solar system as a reason. Thus, the view is that carrying out solar system studies has a good scientific basis. In addition, such studies may lead to a better understanding of the origin of life without making the possible presence of life a reason itself, which would be circular. Similarly, this could be applied to the study of exoplanets. A theme that traverses six decades since its inception is that several molecules including CH4 and NH3 were present that gave rise to the origin of life on the primitive Earth. It is repeatedly stated that life arose under as yet unidentified and unconfirmed conditions [28].

What are the conditions needed for the synthesis and assembly of origin of life components including membranes, proteins, enzymes, ribosomes, lipids, carbohydrates, scaffolds, chirality, diasteriomer etc., let alone of various RNAs and DNA. The extreme conditions under which one currently finds Archaea are the result of several Gyrs of Darwinian evolution and adaptation. However, the origin of life under such hostile conditions may be questionable. Such considerations should include thermodynamics, kinetics, heat, UV light, impacts, electrical discharge, turbulence, entropy, and radioactivity. Therefore, we suggest that to abrogate the null hypothesis requires experimental data to demonstrate that life could occur under any well-defined extreme conditions, even given the existence of Archaea under such conditions, today.

Another issue that has increased significance, is the accumulating huge quantities of data that need analysis. Many types of statistics are needed to address this problem including Bayesian statistics and Markov chains, and others [29, 30, 31].

It would be a statistician's nightmare to attempt to test so many hypotheses, correlate massive wide-ranging data, to produce quantitative statistical weighted levels of significance, and to estimate strictly quantitative probabilities. The huge amount of data that has been amassed during the last six decades and continues to be produced on an expanding scale, require new technologies including AI and quantum computers.

## Postscript

News from another moon of Saturn's, Enceladus, was just reported while this article was in press. Molecules that included carbon and hydrogen were detected above Enceladus with molecular weights upto approximately 200 daltons. This was proposed as a possible biosignature in support of the presence of life [32]. As a concrete example of a molecule containing carbon and hydrogen, a hydrocarbon chain, which has a molecular weight of 198 daltons, could be H-(CH2)14-H.

A very significant problem is that NASA purposefully crashed its exploratory spacecraft 'Cassini'. That is an absurd and dangerous action, because: 1. Data from the spacecraft would have been retrieved by future missions to the Saturn system; 2. There is the unambiguous danger of contaminating Enceladus with terrestrial microorganisms, if indeed Enceladus is in the Goldilocks zone.

This objection and caveat applies to all the solar system objects to which governments and corporations have sent and are sending such probes and rovers: are they really property sterilized to prevent contagions of the places that we are exploring? There is a significant element of doubt, due to the difficulty of the sterilizing process for the intricate machinery that is sent. Requirements and warnings need to be instituted that prevent contamination of the solar system with terrestrial microbes. Just leaving the choices upto each public or private institution to decide, is unwelcome.

## Conflict of Interest

The author reports no conflicts of interest.

## References

[1] https://arxiv.org/pdf/0911.2936.pdf.

[2] https://en.wikipedia.org/wiki/Exoplanet.

[3] http://phl.upr.edu/projects/habitable-exoplanets-catalog.

[4] Wordsworth RD. (2016). Earth and Planetary Science Letters..

[5] Cavicchioli R. (2011). Nat Rev Microbiol..

[6] Williams TA (2013). Nature..

[7] http://www.plb.ucdavis.edu/courses/bis/1c/text/Chapter19nf.pdf.

[8] Vik DR (2017). Peer J..

[9] Sagan C, Chyba C. (1997). Science..

[10] Sagan C, Mullen G. (1972). Science..

[11] http://www.sciam.com.

[12] Krasnopolsky VA. (2006). Planetary and Space Science..

[13] Webster CR (2018). Science..

[14] Sekine Y (2003). J. Geophys. Res..

[15] Pearce BKD, Pudritz RE. (2015). The Astrophysical Journal..

[16] https://www.scientificamerican.com/article/methane-onmars-titan/.

[17] Sagan C. (1977). Nature..

[18] Wordsworth RD. (2016). Annual Review of Earth and Planetary Sciences..

[19] Wordsworth RD (2017). Geophys Res Lett..

[20] Miller SL, Urey HC. (1959). Science..

[21] http://www-ramanathan.ucsd.edu/files/pr89.pdf.

[22] Arney GN (2017). Astrobiology..

[23] Foucher F (2017). Life..

[24] Lineweaver CH, Davis TM. (2002). Bioastronomy Life Among the Stars ASP Conference Series. ArXiv:astrophys/0209385v1.

[25] Lineweaver CH, Davis TM. Does the rapid appearance of life on Earth suggest that life is common in the universe? ArXiv:astrophys/0205014v2.

[26] Anderson DL. (2010). New theory of the Earth. Cambridge Univ. Press (NY, NY)..

[27] Valley JW (2002). Geology.

[28] Abelson PH. (1966). Chemical events on the primitive Earth. PNAS (USA)..

[29] Rosner B. (2000). Fundamental of biostatistics. Fifth edition. (Duxbury Publ. Pacific Grove, CA).

[30] Duncan RC (1983). Introductory biostatistics for the health sciences. Second edition. (Delmar Publ. Albany, NY).

[31] Rozanov YA. (1977). Probability theory. (Dover Publ. NY, NY).

[32] Postberg F (2018). Nature..

